# Vitamin D Decreases Susceptibility of CD4^+^ T Cells to HIV Infection by Reducing AKT Phosphorylation and Glucose Uptake: A Bioinformatic and In Vitro Approach

**DOI:** 10.3390/biom15030432

**Published:** 2025-03-18

**Authors:** John D. Loaiza, Jose Fernando Gómez, Daniel Muñoz-Escudero, Sandra M. Gonzalez, Timothy Kyle Eubank, Maria T. Rugeles, Ana Lucía Rodríguez-Perea, Wbeimar Aguilar-Jimenez

**Affiliations:** 1 Grupo Inmunovirología, Facultad de Medicina, Universidad de Antioquia UdeA, Medellín 050010, ANT, Colombia; 2 Sexually Transmitted and Blood-Borne Infections Division at JC Wilt Infectious Diseases Research Centre, National Microbiology Laboratory Branch, Public Health Agency of Canada, Winnipeg, MB R3E 3L5, Canada

**Keywords:** HIV, vitamin D, glucose uptake, Boolean modeling, protein kinase B, AKT signaling, anti-HIV mechanisms, HIV-1 replication

## Abstract

Activated immune cells are highly susceptible to human immunodeficiency virus (HIV) infection. Vitamin D (VitD) induces antimicrobial responses and reduces cellular activation. We investigated VitD effects on HIV-1 replication, glucose uptake, and gene regulation using computational and in vitro approaches. CD4^+^ T cells from healthy male donors were treated with VitD and infected with HIV-1. After 72 h, p24 protein was measured to assess viral replication. VitD effects on anti- and pro-HIV genes were analyzed by a Boolean network model based on curated databases and the literature. CCR5 and CXCR4 coreceptor expression, AKT phosphorylation, and glucose uptake were evaluated by flow cytometry, and expression of some model-identified genes was quantified by qPCR. VitD reduced p24 by 53.2% (*p* = 0.0078). Boolean network modeling predicted that VitD upregulates antiviral, migration, and cell-differentiation related genes, while downregulating genes related to cellular activation, proliferation, glucose metabolism, and HIV replication, notably *AKT1, CCNT1, SLC2A1, HIF1A,* and *PFKL*. In vitro, VitD reduced AKT phosphorylation by 26.6% (*p* = 0.0156), transcription of *CCNT1* by 22.7% (*p* = 0.0391), and glucose uptake by 22.8% (*p* = 0.0039) without affecting classic antiviral genes or coreceptor expression. These findings suggest an anti-HIV effect of VitD, mediated through AKT and glucose metabolism downmodulation, both involved in cell activation and HIV-1 replication.

## 1. Introduction

HIV/AIDS remains one of the most pressing public health challenges globally. Approximately 39.9 million people worldwide are currently living with HIV, and the development of a cure or effective vaccine remains elusive [1].

HIV primarily targets activated CD4^+^ T cells that express high levels of coreceptors CCR5 and CXCR4, which are essential for viral entry. Activated CD4^+^ T cells undergo rapid proliferation, with an elevated demand for energy and metabolic output, generating favorable conditions for HIV replication. Several steps throughout the viral cycle, including entry, latency, and replication, depend on the activation of diverse cellular signaling pathways in CD4^+^ T cells. Among these, the PI3K/AKT/mTOR pathway plays a critical role in cell survival, growth, proliferation, and glucose metabolism. Notably, HIV preferentially infects cells with enhanced glycolytic activity and increased expression of the glucose transporter 1 (Glut1) [2,3,4]. This preference could explain the resistance to viral replication exhibited by cells with an immunoquiescent phenotype [5,6,7], a trait widely observed in cells from HIV-exposed seronegative individuals (HESNs) who naturally resist HIV infection [8].

Vitamin D (VitD) has emerged as a promising immunomodulator due to its capacity to reduce activation of T cells [9], decrease inflammation [10,11], induce production of antimicrobial peptides [12,13], and suppress HIV replication in peripheral blood mononuclear cells (PBMCs) exposed to viral particles in vitro [14,15,16,17].

Our previous findings further suggest that VitD may contribute to the resistance mechanisms of HESNs by upregulating the expression of anti-inflammatory cytokine IL-10 and antimicrobial defensins [17,18]. Additionally, VitD has been shown to downregulate the expression of PI3K, AKT, and mTOR in a cancer model [19] and modulate glucose metabolism via Akt-mediated signaling pathways in mice [20]. However, whether VitD can modulate HIV infection by targeting this signaling pathway remains unclear. This pathway has been proposed as an innovative therapeutic target against HIV [21].

In this study, we investigated the effects of VitD on HIV-1 replication in vitro in CD4^+^ T cells and in silico through a Boolean network model to predict whether VitD modulates host genes associated with HIV replication. Selected candidate pro- and anti-HIV genes and their associated cellular processes were validated in vitro using flow cytometry and qPCR. Our findings reveal that VitD reduces HIV replication, potentially by downregulating AKT activity and glucose metabolism, both of which are critical for T-cell activation and HIV-1 replication.

## 2. Materials and Methods

### 2.1. Study Population and Sample Preparation for In Vitro Assays

In vitro assays were performed using blood samples collected from eight healthy, HIV-negative male donors. Exclusion criteria included the use of immunosuppressive, anti-inflammatory, or anticoagulant medications, dietary VitD supplementation, recent acute febrile episodes, and chronic illnesses. All participants provided written informed consent prior to enrollment. The study complied with the principles of the Declaration of Helsinki and was approved by the Ethics Committee of Universidad de Antioquia (ACT-008-2016).

### 2.2. CD4^+^ T Cell Isolation

The PBMCs were isolated using Ficoll-Histopaque^®^-1077 (Sigma-Aldrich, Darmstadt, Germany) and resuspended in X-VIVO 15 media (Lonza, Basel, Switzerland), a fetal bovine serum (FBS)-free and VitD-free medium ideal for VitD-focused studies. Viability and cell counts were determined with 1% Trypan blue (Sigma-Aldrich, Darmstadt, Germany).

CD4^+^ T cells were purified by negative selection with immunomagnetic beads (Miltenyi Biotec, Bergisch Gladbach, Germany). Purity (>85%) and viability (>95%) were confirmed by flow cytometry using anti-CD3-Alexa Fluor™ 700, anti-CD4-Pacific Blue™ antibodies, and Fixable Viability Dye-eFluor™ (Invitrogen™, Waltham, MA, USA) (Figure S1).

### 2.3. CD4^+^ T Cell Infection

CD4^+^ T cells were seeded at a density of 3.0 × 10^5^ cells per well in 96-well plates containing 200 μL of X-VIVO 15 medium. Cells were stimulated with 8 μg/mL PHA (Sigma-Aldrich, Darmstadt, Germany) and 50 IU/mL IL-2 (R&D systems, Minnneapolis, MN, USA) and treated with either the active form of VitD (calcitriol; Sigma-Aldrich, Darmstadt, Germany) at 5 nM (a concentration found in serum after calcitriol supplementation [22,23] and in dendritic cells supernatants ex vivo [24,25]) or with 0.01% ethanol (EtOH) as the vehicle control. Unstimulated cells were included as controls for cytometric analysis. Cultures were incubated at 37 °C with 5% CO_2_ with media being changed every 48 h while maintaining VitD and EtOH concentrations.

Forty-eight hours post-activation, 7.5 × 10^5^ to 1.6 × 10^6^ CD4^+^ T cells were transferred to 1.5 mL vials and infected with 90 µL of X4-tropic HIV-1 (equivalent to 6.5 ng p24) obtained from H9-HTLV-IIIB cell line supernatants (ATCC-CRL-8543). Cells were incubated with the virus for 30 min at 37 °C with 5% CO_2_, followed by spinoculation at 1200× *g* for 2 h at room temperature [26]. After washing with PBS (Sigma-Aldrich, Darmstadt, Germany), cells were cultured in X-VIVO 15 media (Lonza, Basel, Switzerland) with their respective VitD or EtOH treatments for 72 h. Viral replication was assessed by measuring p24 protein using an HIV-1 p24 ELISA kit (Xpress Bio, Frederick, MD, USA).

### 2.4. Data Collection and Boolean Model Design for Bioinformatic Simulations

A gene regulatory network was constructed to model molecular dynamics triggered by VitD and HIV infection. Data were sourced from curated databases (STRING version 12.0; TRRUST version 2 [27], the HIV-1, Human Protein Interaction Database [28]), and the primary literature (Supplementary Materials File S1). The network’s nodes represent genes, while edges denote regulatory interactions, classified as activation or inhibition. Activated or repressed genes during VitD or HIV infection were treated as individual nodes. Non-conclusive interactions, such as contradictory outcomes across databases (e.g., simultaneous activation and inhibition), were resolved using additional references or excluded if no consensus was reached. The complete network, comprising 1302 nodes and 4932 interactions (Table S1), was reduced using Net-Synthesis [29] to generate a sparse yet functional model (Figure S2) that could enhance interpretability and computational efficiency. Simplified interactions were used to define the logical dependencies between genes (AND, OR, NOT), and were modeled using Boolean formalism with the Python library BooleanNet [30]. The state of each node at time t + 1 was determined by Boolean expressions combining the states of its regulators at time t. For instance, the regulatory logic of the transcription factor *E2F1*, activated by *NR4A1* and repressed by *CCNA1*, *E2F6*, and *E2F7*, could be expressed as

E2F1 = *not* CCNA1 *or not* E2F6 *or not* E2F7 *or* NR4A1

In the asynchronous setting, each node is updated exactly once per time unit in a specified order, reflecting the progression of regulatory events. At the start of the simulation, nodes were initialized as ON or OFF based on predefined rules. For example, HIV presence (ON or OFF) was simulated by setting the initial states of a specific set of genes according to established rules (Supplementary Materials File S2). After 40 simulation steps, the activation or deactivation states of all genes in the network were visualized in a heat map (Figure S3). This comprehensive view of the network enabled the selection of pro- and anti-HIV genes modulated by VitD for further analysis based on their relevance in the model and in the literature.

### 2.5. Assessment of AKT Phosphorylation and Coreceptors Expression

Purified CD4^+^ T cells were seeded at a density of 4.0 × 10^5^ cells per well in 96-well plates with X-VIVO 15 medium. Cells were treated for 16 h with VitD, EtOH, or 15 µM Miltefosine (Abcam, Cambridgeshire, UK), a known Akt inhibitor [31,32], as a negative control. The cells were then stimulated with 100 nM Calyculin A (Invitrogen™, Waltham, MA, USA), a strong inducer of Akt phosphorylation, in the presence of their respective treatments for 30 min. Cells were harvested for phospho-Akt staining. An unstimulated control was included to establish the basal threshold of Akt phosphorylation.

Cells were stained with Live/Dead Fixable Yellow Dead Cell Stain (Invitrogen™, Waltham, MA, USA), anti-CD3-Alexa Fluor 700 (eBioscience™, San Diego, CA, USA), anti-CD4-V450 (BD™, Franklin Lakes, NJ, USA), anti-CCR5-APC-Cy7 (eBioscience™, San Diego, CA, USA), and anti-CXCR4-PE-Cy5 (BD™, Franklin Lakes, NJ, USA) at 4 °C for 30 min. Stained cells were further fixated and permeabilized using FoxP3/transcription factor staining buffer (eBioscience™, San Diego, CA, USA) according to the manufacturer instructions and intracellularly labeled with anti-phospho-AKT(Ser473)-PE (R&D Systems, Minnneapolis, MN, USA) at 4 °C for 30 min. At least 100,000 events were acquired on an LSR Fortessa X-20 cytometer (BD) and analyzed using FlowJo V10.9.0 (BD™, Franklin Lakes, NJ, USA). Compensation for fluorochrome spillover was performed using unstained and single-stained cells.

AKT phosphorylation was quantified by median fluorescence intensity (MFI) (gating strategy shown in Figure S4).

### 2.6. qPCR Gene Expression Assay

To assess the effect of VitD on the expression of host proviral and antiviral genes, RNA was extracted from 8.0 × 10^5^ CD4^+^ T cells treated with either VitD or EtOH following 30 min of stimulation with Calyculin A and using the Direct-zol RNA Miniprep Kit (Zymo Research, Irvin, CA, USA). Complementary DNA (cDNA) was synthesized using the iScript™ cDNA Synthesis Kit (Bio-Rad, Hercules, CA, USA). Quantitative PCR (qPCR) was conducted for analyzing the expression of the following pro- and anti-HIV genes:

SAMHD1 (Fwd: 5′-CTCGCAACTCTTTACACCGTAGA-3′, Rev: 5′-TTTCCTCCAGCACCTGTAATCTC-3′), associated with antiviral activity.

PLD1 (Fwd: 5′-TTCCAAAGTCTCAAACAACAGCC-3′, Rev: 5′-AGCAGAGCGGAGCAACTGT-3′), involved in cellular activation signaling.

ADAM10 (Fwd: 5′-CGGGGATGGGAGGTCAGTAT-3′, Rev: 5′-ACGCTGGTGTTTTTGGTGTA-3′), and CCNT1 (Fwd: 5′-TTCATGGCAACCAACAGCC-3′, Rev: 5′-CCCGTCAGTTGAGACTGGGA-3′), required for viral replication, trafficking, and latency reversal.

VDR (Fwd: 5′-TGCTATGACCTGTGAAGGCTG-3′, Rev: 5′-AGTGGCGTCGGTTGTCCTT-3′) and VitD-target gene CYP24A1 (Fwd: 5′-CGCAAATACGACATCCAGGC-3′, Rev: 5′-AATACCACCATCTGAGGCGT-3′), used as controls for VitD transcriptional regulation.

PGK1 (Fwd: 5′-GTTGACCGAATCACCGACC-3′, Rev: 5′-TCGACTCTCATAACGACCCGC-3′), served as the reference gene for normalization.

The qPCRs were performed using SYBR™ Green Universal Master Mix (Thermo Fisher Scientific, Waltham, MA, USA) in a CFX-96 real-time thermal cycler (Bio-Rad, Hercules, CA, USA).

The thermal cycling conditions were as follows: initial enzyme activation at 95 °C for 10 min, followed by 40 cycles of denaturation at 94 °C for 8 s and annealing at 60 °C for 40 s (except for *SAMHD1* that annealed at 62 °C). Relative expression units of mRNA (RU) were calculated by the ΔCt method [33] using the expression of *PGK1* to normalize the amount of RNA in the samples.

### 2.7. Glucose Uptake Assay

The CD4^+^ T cells infected with HIV, as previously described, were treated with 5 nM active VitD or 0.01% EtOH for 72 h. The medium was replaced after 48 h while maintaining VitD and EtOH concentrations. At the end of the 72 h culture period, the cells were harvested for glucose uptake assays. Cells were centrifuged at 700× *g* for 5 min and subsequently incubated for 3 h at 37 °C, 5% CO_2_ in 200 µL glucose-free and FBS-free RPMI. After incubation, 2 µL of glucose analog 2-(N-(7-Nitrobenz-2-oxa-1,3-diazol-4-yl)Amino)-2-Deoxyglucose (2-NBDG) (Invitrogen™, Waltham, MA, USA) in a 1:40 dilution was added to the cells, and flow cytometry was carried out after a 10 min incubation following the manufacturer’s instructions. The MFI of 2-NBDG was measured using the FITC channel.

### 2.8. Statistical Analysis

Statistical analyses were conducted using GraphPad Prism version 8.0.1 for Windows (GraphPad Software Inc., San Diego, MA, USA). Data differences between VitD and EtOH treatments were analyzed using either the *t*-test or the Wilcoxon test, depending on whether data were normally or non-normally distributed as assessed by the Shapiro–Wilk test. Spearman’s correlation analysis was conducted to determine the correlation between VitD, glucose uptake, and viral particle concentration. A *p*-value < 0.05 was considered statistically significant.

## 3. Results

### 3.1. Effect of VitD on HIV-1 Infection

**Figure 1:**
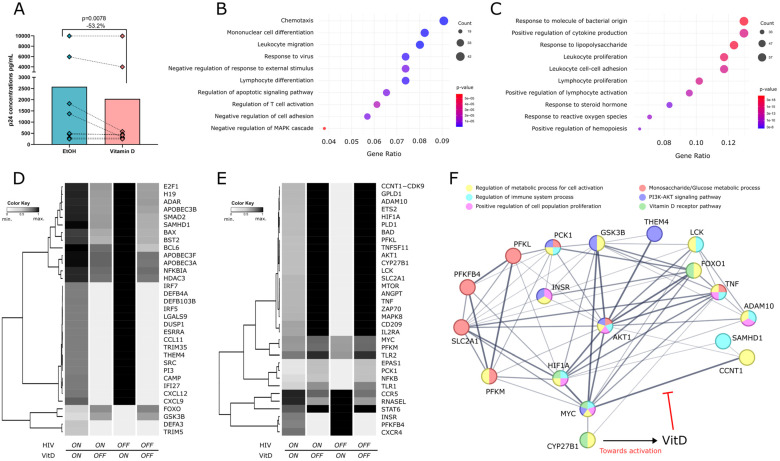
VitD reduces HIV replication in vitro and modulates host gene expression in silico. (**A**) p24 concentrations in CD4^+^ T-cell supernatants 72 h post-infection in the presence of VitD or EtOH (vehicle control). Points represent individual values. Statistical comparisons were performed using Wilcoxon matched pairs signed rank test. (**B**) GO enrichment analysis of genes positively and (**C**) negatively modulated by VitD based on the Boolean network model using publicly available datasets. Dot plots highlight the top enriched GO terms associated with genes upregulated and downregulated in the presence of VitD. Dot plots highlight the top enriched GO terms, with dot size representing the number of genes in each term, colors representing *p*-values and the *x*-axis showing the gene ratio. (**D**) Heatmaps displaying the simulated regulatory behavior of selected antiviral and (**E**) proviral genes in response to VitD under different conditions (VitD ON/OFF and HIV ON/OFF). Rows correspond to individual genes and columns represent the last 20 averaged simulation steps for each condition. (**F**) Undirected interaction network of AKT and other VitD-modulated genes in the Boolean model, with roles in cellular activation, metabolism, and anti-HIV responses. GO terms associated with each protein are color coded, with each color representing a specific biological process or metabolic pathway. Lines indicate confidence levels, ranging from 0.15 to 0.90, with thicker lines representing higher confidence. The network and GO enrichment analyses from the Boolean model network were generated using information available on the STRING Database, incorporating evidence for functional and physical interactions.

To evaluate the impact of VitD treatment on the susceptibility of CD4^+^ T cells to HIV-1 infection, levels of p24 were quantified in the supernatants from VitD- or EtOH-treated cells after 72 h post-infection using an ELISA assay. VitD-treated cells exhibited a significant reduction of 53.2% on the levels of p24 in 7 out of 8 individuals, compared to EtOH-treated cells (Median: 438.135 pg/mL vs. 936.235 pg/mL, respectively, *p* = 0.0078) (Figure 1A).

### 3.2. Boolean Modeling of VitD Effects in Regulating Host Pro- and Antiviral Genes

Given the observed reduction in HIV infection of purified CD4^+^ T cells following VitD treatment in vitro, we employed a Boolean network model using publicly available data to investigate the regulatory effects of VitD on the expression of host proviral and antiviral genes. Gene Ontology (GO) enrichment analysis, based on the Boolean network model, revealed that VitD upregulates genes involved in antiviral responses, leukocyte migration, T-cell differentiation, and negative responses to stimuli, while primarily downregulating genes associated with cellular activation and proliferation (Figure 1B,C). This analysis further revealed dynamic shifts occurring in the regulation of antiviral and proviral genes under four conditions: the presence (ON) or absence (OFF) of both HIV and VitD. A global visualization of all network genes is presented in Figure S3, provided as a vector-scalable PDF to enable a clear reading of gene names on the *y*-axis and zooming into specific regions of interest. To identify true hits (proviral and antiviral genes), we averaged all 40 simulation steps to reduce variability and applied a fold-change-based method. Genes with a fold-change greater than 1 were considered more active under Vitamin D treatment (e.g., FC = 1.4 indicates a 40% increase), while those with a fold-change less than 1 were less active (e.g., FC = 0.7 indicates a 30% decrease). Significant hits are visually distinguished in the heatmap by pronounced color transitions, whereas pseudo-hits display subtler changes. A subset of 33 antiviral and 33 proviral genes modulated by VitD is shown in Figure 1D,E. Specifically, antiviral genes (Figure 1D) were selected based on their confirmed anti-HIV roles or enrichment in GO terms related to antiviral responses, cellular migration, and negative regulation of activation, whereas proviral genes (Figure 1E) were chosen for their established roles in facilitating HIV infection or enrichment in GO terms associated with proliferation, activation, and metabolic processes.

Notably, several antiviral genes, including *SAMHD1*, *APOBEC* family genes, defensins, *IRF7*, the interferon-inducible factor *tetherin* (*BST2*), and *CXCL12*, showed increased expression when VitD was ON, but were downregulated in the presence of HIV, with the exception of *SAMHD1* and *APOBEC* genes, which remained largely unaffected by HIV (Figure 1D). In contrast, proviral genes such as *AKT1*, *PLD1*, *ADAM10*, *TNF*, *LCK*, *SLC2A1*, and *CCNT1*, which are implicated in cellular activation, latency maintenance, and viral replication, were upregulated in response to HIV but consistently downregulated by VitD, regardless of HIV presence (Figure 1E). The model also predicts that VitD induces expression of the HIV coreceptor CXCR4 and, to a lesser extent, CCR5, both essential for viral entry.

Interestingly, VitD downregulates several genes related to glucose metabolism and the AKT signaling pathway, including *AKT1*, *GSK3B*, *MYC*, *SLC2A1*, *HIF1A*, *FOXO*, *PCK1*, *PFKM*, and *PFKL*, while upregulating *THEM4*, *INSR*, and *PFKFB4* (Figure 1D,E). Indeed, an interaction network of the above-mentioned VitD-modulated genes created using STRING database V12.0 [34] (available at https://string-db.org; accessed on 30 November 2024) shows the interplay and connections among AKT, antiviral, and glucose metabolism genes.

Other genes involved in glucose metabolism, such as *G6PD* and *GAPDH*, remained unchanged in our simulations (Figure S3).

### 3.3. VitD Reduces AKT Phosphorylation, CCNT1 Expression and Glucose Uptake In Vitro

**Figure 2:**
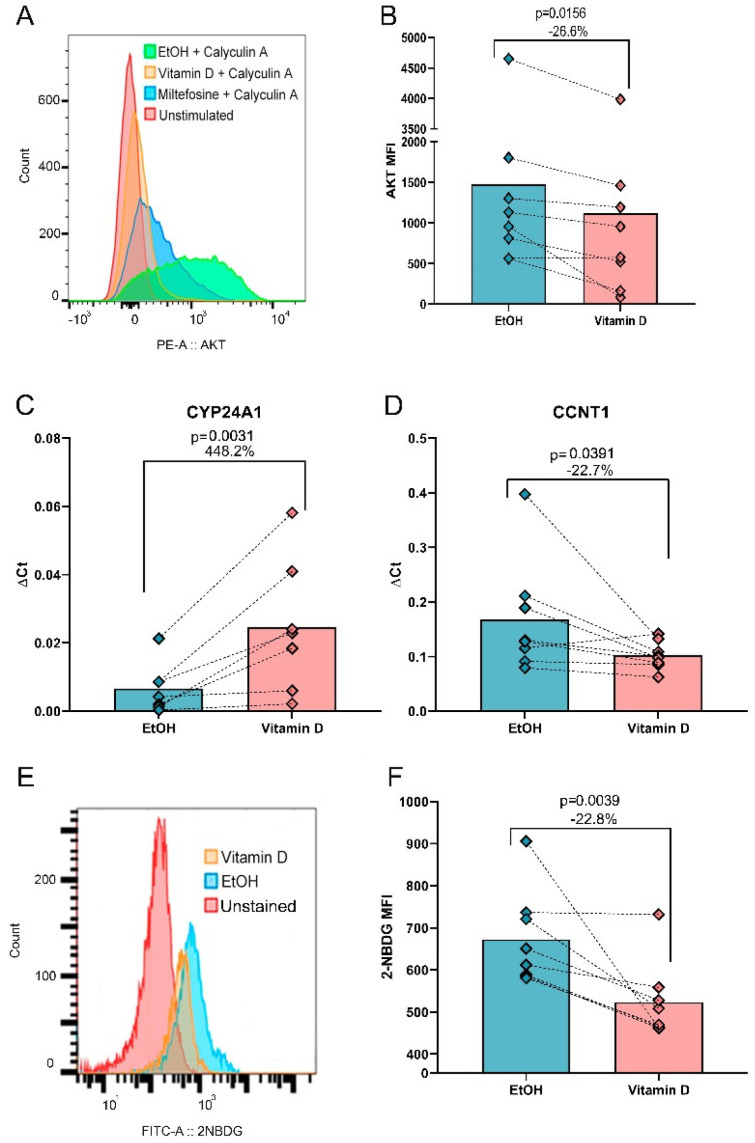
VitD reduces AKT phosphorylation, modulates gene expression, and decreases glucose uptake in CD4^+^ T cells in vitro. (**A**) Representative flow cytometry histogram showing the MFI of pAKT upon 30 min of calyculin stimulation, either under VitD treatment with EtOH as a vehicle control, or miltefosine as a negative control (**B**) MFI of pAKT upon VitD stimulation versus vehicle control (EtOH). (**C**) Effect of VitD on gene regulation by qPCR. *CYP24A1*, a gene known to be regulated by the active form of VitD was used as a control. (**D**) Expression of proviral gene *CCNT1* decreased after VitD stimulation. (**E**) Flow cytometry histogram showing the MFI of 2-NBDG under different experimental conditions (**F**) MFI of 2-NDBG uptake upon VitD stimulation versus vehicle control. Wilcoxon matched pairs signed rank test was used for all statistical comparisons.

Based on the bioinformatic predictions, we investigated the effects of VitD on key pathways identified in silico, including AKT phosphorylation, glucose uptake, and the expression of antiviral and proviral genes. Flow cytometry analysis confirmed that VitD significantly reduced the MFI of phosphorylated AKT (pAkt) by 26.6% compared to the EtOH control (MFI: 766 vs. MFI: 1044, *p* = 0.0156, Figure 2A,B), after validating the assay using Calyculin A (an AKT phosphorylation inducer) and miltefosine (an AKT inhibitor). To validate the predicted gene regulation by VitD, we analyzed the mRNA expression of *SAMHD1*, *PLD1*, *ADAM10*, and *CCNT1*, which are critical genes for HIV-1 replication [35], along with *VDR* and *CYP24A1* as VitD-regulated genes.

As expected, the expression of *CYP24A1,* a well-established VitD-induced gene [36], increased by 448.2% (*p* = 0.0156, Figure 2C). Notably, VitD reduced the expression of *CCNT1* expression by 22.7% (*p* = 0.0391, Figure 2D). However, the expression of the remaining four genes analyzed by qPCR, and HIV coreceptors CXCR4 and CCR5 measured by flow cytometry, showed no significant changes (Figures S5 and S6).

Given the downregulation of PFKL and AKT observed in silico, we further investigated glucose uptake using the fluorescent glucose analog 2-NBDG. VitD-treated cells exhibited a 22.8% reduction in glucose uptake compared to EtOH-treated controls (MFI: 487.5 vs. MFI: 634.5, *p* = 0.0039, Figure 2E,F). However, no significant linear correlation was found between glucose uptake and p24 levels (r = 0.04657, *p* = 0.8785), suggesting that VitD-mediated effects on glucose metabolism and HIV replication occur through distinct pathways. These effects are independent of cell viability since 90% of cells were alive and no differences between VitD and EtOH treatments were found (Figure S7).

## 4. Discussion

Our study confirmed that VitD decreases HIV-1 infection in CD4^+^ T cells. This finding aligns with prior reports from our group and others using various in vitro models [14,15,17,37] and in vivo approaches [16]. These anti-HIV effects are thought to be mediated by the induction of anti-inflammatory profiles and reduced T-cell activation [9,10,11,17,18].

We demonstrated that AKT, also known as protein kinase B (PKB), a serine/threonine kinase essential for regulating metabolism, proliferation, and cell survival [38], is downregulated by VitD, as shown through both in silico and in vitro assays. The functionality of VitD in our experimental conditions was validated by measuring the transcriptional expression of *CYP24A1*, a well-established VitD-responsive gene, which was significantly upregulated, consistent with previous findings [36]. Our Boolean model identified AKT as a central regulator of VitD-regulated genes involved in metabolism, growth, cellular proliferation, and HIV replication. In CD4^+^ T cells, AKT is phosphorylated upon antigenic and co-stimulatory signals, activating downstream targets such as GSK3-β, mTORC1, CREB, Foxo, and NF-κB, which regulate activation, survival, and differentiation in these cells [38]. Notably, VitD-induced upregulation of THEM4, a known inhibitor of AKT [39], could explain the AKT inhibition observed in our model. This has been previously demonstrated in macrophages where VitD inhibited expression of *THEM4* independently of LPS stimulus due to the presence of a negative VitD responsive element within the *THEM4* gene [40].

The energy metabolism of T cells is intrinsically linked to their activation state. Resting T cells primarily depend on oxidative phosphorylation and fatty acid oxidation, whereas activation triggers metabolic reprogramming toward glycolysis to meet the energy demands of effector functions [41]. Because HIV replication requires activated T cells, increased glucose uptake and glycolysis efficiently promote viral replication [42,43].

AKT modulates glucose metabolism by targeting receptors and enzymes that promote aerobic glycolysis, ensuring sufficient energy supply for cell activation [44]. Consistently, our Boolean model predicted that VitD reduced *SLC2A1*, which encodes GLUT1, a key glucose transporter regulated by AKT. GLUT1 plays a crucial role in maintaining CD4^+^ T-cell activation and is a marker of poor prognosis in HIV infection [45] and cancer [46]. In HIV-infected individuals, GLUT1 expression is upregulated in CD4^+^ T cells, correlating with increased glucose metabolism, cellular activation, and effector functions. This upregulation persists despite antiretroviral therapy and is associated with loss of CD4^+^ T cells and disease progression [45]. Given the association of GLUT1 expression with immune activation and disease severity, this glucose transporter could serve as a potential prognostic marker for both HIV and cancer [45,46].

Remarkably, our in vitro assays demonstrated that VitD reduces glucose uptake, mirroring findings in breast cancer cells [47]. VitD also decreased the expression of glycolytic enzymes phosphofructokinase (PFK), the main regulatory enzyme of glycolysis, and phosphoenolpyruvate carboxykinase (PCK1), a rate-limiting enzyme in gluconeogenesis. Both enzymes are predicted to interact with AKT in our interaction network. Furthermore, overexpression of PFK and PCK1 in T cells has been linked to increased glycolytic flux and enhanced effector functions [48,49]. Thus, VitD may reduce T-cell activation by downregulating glycolytic pathways. Interestingly, VitD upregulated PFKB4, an isoform of phosphofructokinase 2 (PFK2) with dual kinase and phosphatase functions. While the kinase activity of this enzyme could promote glycolysis, its phosphatase activity may promote the pentose phosphate pathway and NADPH generation, potentially reducing oxidative stress and enhancing survival in the absence of glycolysis, as observed in cancer [50].

In addition to regulating T-cell activation, VitD supplementation has been shown to increase circulating regulatory (Treg) cells and positively modulate their suppressive phenotype in both healthy individuals and patients with inflammatory disease [51]. The PI3K/AKT/mTOR pathway, reduced by VitD in our bioinformatic model, integrates glucose metabolism and T-cell signaling, influencing T-cell fate. In peripheral CD4^+^ T cells, this pathway is essential for differentiation into Th1, Th2, and Th17 effector subsets [52], and it might also play a significant role in Treg development, since the phosphorylation status of AKT may lead to alternative T-cell profiles. It has been observed that a low TCR signal strength leads to low AKT/mTOR signaling, resulting in induction of Treg cells in a murine model [53]. In addition, reduced glucose uptake, Glut1 expression and glycolytic activity are pivotal in promoting Treg induction. Glut1-mediated glucose uptake and expression of glycolytic cellular machinery are required for effector CD4^+^ T-cell activation, subsequent metabolic reprogramming and clonal expansion. In contrast, Tregs rely on alternative metabolic pathways, such as fatty acid oxidation, and exhibit decreased levels of GLUT1 expression. This reduction is regulated by the transcription factor FOXP3, aligning with Tregs’ diminished reliance on glycolysis [54,55,56].

Our model also predicts that VitD downregulates GSK3B, a regulatory glycogen synthase kinase typically inhibited by the PI3K/AKT pathway [57,58]. Reduction of GSK3B has been shown to induce Treg [59], and the isoforms of this kinase have distinct effects, either enhancing or attenuating T-cell activation [57]. Additionally, VitD downregulated c-Myc, a transcription factor downstream of AKT phosphorylation that is crucial for CD4^+^ T-cell development, differentiation, and activation [60]. c-Myc induces the expression of GLUT1, PFK, and other glycolytic proteins, and its inhibition abrogates T-cell growth and proliferation [60]. c-Myc also regulates the cell cycle by upregulating cyclins such as CCNA2, which promote G1/S and G2/M transitions. Our model predicts that VitD reduces CCNA2 expression, likely through c-Myc downregulation [61].

Furthermore, our model predicts that VitD reduces HIF1α, a transcription factor that regulates oxygen levels and hypoxic adaptive metabolic responses. Decreased HIF1α expression in CD4^+^ T cells inhibits glycolytic gene expression, including GLUT1 and rate-limiting enzymes such as hexokinase 2 (HK2) and pyruvate kinase myoisozyme 2 (PKM2) [44].

Although the model predicts that VitD promotes some pro-glycolytic nodes, such as the insulin receptor (INSR), which activates Glut1 in a PI3K-AKT-dependent manner [62], the net reduction in Glut1 predicted by the model could be attributed to the downregulation of major nodes such as AKT and HIF1A. These nodes control glycolysis and glucose transporter trafficking and expression, ultimately limiting glucose uptake, as confirmed in vitro, which is essential for HIV-infected cells [45,63].

These findings suggest that VitD may reprogram cellular metabolism to a state less favorable for viral replication through downregulation of AKT, thereby reducing the glycolytic pathways essential for cellular activation and therefore, limiting HIV infection. However, the complex interplay between INSR, GSK3B, and AKT observed in our model underscores the need for further studies to fully elucidate these regulatory pathways and their therapeutic potential.

In addition to its metabolic effects, VitD seems to restrict viral replication independently of cellular metabolism. We observed that VitD downregulates Cyclin-T1 both in silico and transcriptionally in vitro. Cyclin-T1, encoded by CCNT1, is the regulatory subunit of positive transcription elongation factor b (P-TEFb) [64], which is recruited by the HIV-1 Tat protein during viral genome replication. P-TEFb phosphorylates RNA polymerase II, enhancing transcriptional elongation and viral replication [65,66]. Since Cyclin-T1 expression is increased in activated lymphocytes via AKT activation [66,67], VitD may induce an antiviral state by reducing kinase cascades critical for both HIV replication and cellular activation.

VitD also modulates known HIV restriction factors in silico, such as RNA-editing APOBEC proteins, antiprotease Elafin (*PI3*) and SAMHD1, a deoxynucleotide triphosphate hydrolase that reduces intracellular dNTP pools [35,68]. Whereas our previous results support a potential positive effect of VitD on the expression of APOBEC proteins and Elafin in mononuclear cells [17], there is little evidence available describing the effects of VitD on the expression of SAMHD1, a protein that enhances antiviral response by reducing cellular activation [69]. Although our model predicted that VitD increases SAMHD1, no significant increase in SAMHD1 mRNA was observed in response to VitD in our in vitro assays, requiring caution when interpreting the model findings.

In contrast with the antiviral state promoted by VitD, our model predicts the upregulation of HIV coreceptors CXCR4 and CCR5, essential for viral entry. Interestingly, CCR5 restricts aerobic glycolysis in memory CD4^+^ T cells and organizes TCR nanoclusters, reducing the T-cell activation threshold [70]. VitD’s potential upregulation of CXCR4 could result from increased expression of its ligand, stromal cell-derived factor 1 (SDF1 or CXCL12), also predicted by the model, which has been observed to impede HIV entry, protecting against infection [71]. However, we observed no changes in protein levels of these coreceptors in our in vitro assays, emphasizing the need for further research to understand the implications of these findings.

Our study has several limitations. First, discrepancies between experimental and computational results may stem from the heterogeneity of data used in the bioinformatic model, which relied on public databases, whereas our in vitro experiments focused on primary CD4^+^ T cells. Future studies incorporating T-cell-specific datasets, along with additional in vitro and ex vivo experiments, are needed to refine predictions and validate their biological relevance [72]. Second, the binary nature of gene expression modeling simplifies gene activity, potentially overlooking critical regulatory nuances. Gene interactions often rely on precise timing and sequential expressions that Boolean functions cannot fully capture. Third, we faced challenges in constructing a comprehensive Boolean model that fully represents the HIV-host gene regulatory network. The available HIV-host protein interaction databases and literature primarily focus on individual or small groups of genes, limiting our ability to identify specific genes that are necessary or sufficient for HIV replication. While Boolean modeling offered a framework to explore interactions among 227 pro- and anti-HIV host genes under VitD stimulation, further investigation with more dynamic and integrative modeling approaches is warranted.

Finally, it is important to acknowledge the limitation of exclusively using male donors for our in vitro studies. Women were excluded from this study to eliminate the variability of cellular responses related to fluctuating estrogen levels, which cause differential T-cell subset values and cytokine expression profiles [73,74].

## 5. Conclusions

Despite these limitations, our in vitro assays, albeit constrained by a smaller sample size, demonstrated the beneficial effects of VitD in reducing p24 concentration, AKT phosphorylation, and glucose uptake, suggesting that VitD’s capacity to decrease cellular activation may result in reduced HIV infection. It is worth noting that viral replication was measured only at 72 h, an appropriate timeframe to assess viral replication. In future studies, it could be beneficial to examine other time points to determine if the effect of VitD on viral replication is sustained over time. Moreover, VitD appears to restrict viral replication by modulating the transcription of antiviral and metabolic genes. Collectively, these findings highlight the potential of VitD as an adjunctive therapeutic strategy in HIV management. Future studies should include larger sample sizes and further explore the effects of VitD on glucose transporter expression and glycolytic pathways to further elucidate its mechanism of action.

## Data Availability

The original contributions presented in this study are included in the article and Supplementary Material. Further inquiries can be directed to the corresponding author(s). Input data for our computational models were derived from the following resources available in the public domain: NCBI HIV-1-Human Protein Interaction Database, available at http://www.ncbi.nlm.nih.gov/genome/viruses/retroviruses/hiv-1/interactions (accessed on 29 January 2025), STRING Version 12.0, available at https://string-db.org, and TRRUST Version 2.0, available at https://www.grnpedia.org/trrust (accessed on 29 January 2025). Our custom BooleanNet script is available under File S1 for public use. For proper script execution, the file extension format must be changed from .txt to .py.

## Supplementary Materials

The following supporting information can be downloaded at https://www.mdpi.com/article/10.3390/biom15030432/s1, Table S1: Complete network and interactions. File S1: HIV interaction database. File S2: Custom BooleanNet script and rules for simulating the effect of VitD and HIV. Figure S1: Purity and viability assessment of CD4^+^ T Cells. Figure S2: Simplified gene regulatory network of interactions relevant to HIV infection. Figure S3: Heatmap of node states across 40 simulation steps in the gene regulatory network. Figure S4: Gating strategy to determine the effect of VitD on AKT phosphorylation. Figure S5: qPCR results of genes not modulated by VitD. Figure S6: Effect of VitD on the expression of CXCR4 and CCR5 coreceptors. Figure S7: Viability of CD4^+^ T cells under VitD and EtOH treatments.

## Abbreviations

The following abbreviations are used in this manuscript:

2-NBDG	2-(N-(7-nitrobenz-2-oxa-1,3-diazol-4-yl)amino)-2-deoxyglucose
AIDS	Acquired immunodeficiency syndrome
AKT, PKB	Protein kinase B
APOBEC	Apolipoprotein B mRNA-editing enzyme, catalytic polypeptide
BST2	Tetherin
CCR5	C-C chemokine receptor type 5
CD	Cluster of differentiation
cDNA	Complementary DNA
CO2	Carbon dioxide
CXCR4	C-X-C chemokine receptor type 4
DNA	Deoxyribonucleic acid
dNTP	Deoxynucleotide triphosphate
ELISA	Enzyme-linked immunosorbent assay
EtOH	Ethanol
FBS	Fetal bovine serum FBS
FOXP3	Forkhead Box P3
Glut1	Glucose transporter 1
GO	Gene Ontology
HESNs	HIV-exposed seronegative individuals
HIV	Human immunodeficiency virus.
HK2	Hexokinase 2
INSR	Insulin receptor
MFI	Median fluorescence intensity
mRNA	Messenger ribonucleic acid
mTOR	Mechanistic target of rapamycin
NADPH	Nicotinamide adenine dinucleotide phosphate
pAKT	Phosphorylated AKT
PBMCs	Peripheral blood mononuclear cells
PCK1	Phosphoenolpyruvate carboxykinase
PFK	Phosphofructokinase
PFK2	phosphofructokinase 2
PHA	Phytohemagglutinin
PI3	Elafin
PI3K	Phosphoinositide 3-kinase
P-TEFb	Positive transcription elongation factor b
qPCR	Quantitative polymerase chain reaction
RNA	Ribonucleic acid
RU	Relative expression units of mRNA
SDF1, CXCL12	Stromal cell-derived Factor 1
TCR	T-cell receptor
Th	Helper T cell
Treg	Regulatory T cell
VitD	Vitamin D

## References

[1] UNAIDS Global HIV & AIDS Statistics—Fact Sheet. https://www.unaids.org/en/resources/fact-sheet.

[2] Palmer C.S., Duette G.A., Wagner M.C.E., Henstridge D.C., Saleh S., Pereira C., Zhou J., Simar D., Lewin S.R., Ostrowski M. (2017). Metabolically active CD4+ T cells expressing Glut1 and OX40 preferentially harbor HIV during in vitro infection. FEBS Lett..

[3] Dickerson J.E., Pinney J.W., Robertson D.L. (2010). The biological context of HIV-1 host interactions reveals subtle insights into a system hijack. BMC Syst. Biol..

[4] Valle-Casuso J.C., Angin M., Volant S., Passaes C., Monceaux V., Mikhailova A., Bourdic K., Avettand-Fenoel V., Boufassa F., Sitbon M. (2019). Cellular Metabolism Is a Major Determinant of HIV-1 Reservoir Seeding in CD4(+) T Cells and Offers an Opportunity to Tackle Infection. Cell Metab..

[5] Gonzalez S.M., Taborda N.A., Feria M.G., Arcia D., Aguilar-Jimenez W., Zapata W., Rugeles M.T. (2015). High Expression of Antiviral Proteins in Mucosa from Individuals Exhibiting Resistance to Human Immunodeficiency Virus. PLoS ONE.

[6] Lajoie J., Juno J., Burgener A., Rahman S., Mogk K., Wachihi C., Mwanjewe J., Plummer F.A., Kimani J., Ball T.B. (2012). A distinct cytokine and chemokine profile at the genital mucosa is associated with HIV-1 protection among HIV-exposed seronegative commercial sex workers. Mucosal Immunol..

[7] Yao X.D., Omange R.W., Henrick B.M., Lester R.T., Kimani J., Ball T.B., Plummer F.A., Rosenthal K.L. (2014). Acting locally: Innate mucosal immunity in resistance to HIV-1 infection in Kenyan commercial sex workers. Mucosal Immunol..

[8] Miyazawa M., Lopalco L., Mazzotta F., Lo Caputo S., Veas F., Clerici M., ESN Study Group (2009). The ‘immunologic advantage’ of HIV-exposed seronegative individuals. AIDS.

[9] von Essen M.R., Kongsbak M., Schjerling P., Olgaard K., Odum N., Geisler C. (2010). Vitamin D controls T cell antigen receptor signaling and activation of human T cells. Nat. Immunol..

[10] Hansdottir S., Monick M.M., Lovan N., Powers L., Gerke A., Hunninghake G.W. (2010). Vitamin D decreases respiratory syncytial virus induction of NF-kappaB-linked chemokines and cytokines in airway epithelium while maintaining the antiviral state. J. Immunol..

[11] Korf H., Wenes M., Stijlemans B., Takiishi T., Robert S., Miani M., Eizirik D.L., Gysemans C., Mathieu C. (2012). 1,25-Dihydroxyvitamin D3 curtails the inflammatory and T cell stimulatory capacity of macrophages through an IL-10-dependent mechanism. Immunobiology.

[12] McMahon L., Schwartz K., Yilmaz O., Brown E., Ryan L.K., Diamond G. (2011). Vitamin D-mediated induction of innate immunity in gingival epithelial cells. Infect. Immun..

[13] Wang T.T., Nestel F.P., Bourdeau V., Nagai Y., Wang Q., Liao J., Tavera-Mendoza L., Lin R., Hanrahan J.W., Mader S. (2004). Cutting edge: 1,25-dihydroxyvitamin D3 is a direct inducer of antimicrobial peptide gene expression. J. Immunol..

[14] Campbell G.R., Spector S.A. (2011). Hormonally active vitamin D3 (1α,25-dihydroxycholecalciferol) triggers autophagy in human macrophages that inhibits HIV-1 infection. J. Biol. Chem..

[15] Coussens A.K., Naude C.E., Goliath R., Chaplin G., Wilkinson R.J., Jablonski N.G. (2015). High-dose vitamin D3 reduces deficiency caused by low UVB exposure and limits HIV-1 replication in urban Southern Africans. Proc. Natl. Acad. Sci. USA.

[16] Stallings V.A., Schall J.I., Hediger M.L., Zemel B.S., Tuluc F., Dougherty K.A., Samuel J.L., Rutstein R.M. (2015). High-dose vitamin D3 supplementation in children and young adults with HIV: A randomized, placebo-controlled trial. Pediatr. Infect. Dis. J..

[17] Aguilar-Jimenez W., Villegas-Ospina S., Gonzalez S., Zapata W., Saulle I., Garziano M., Biasin M., Clerici M., Rugeles M.T. (2016). Precursor Forms of Vitamin D Reduce HIV-1 Infection In Vitro. J. Acquir. Immune Defic. Syndr..

[18] Aguilar-Jimenez W., Zapata W., Caruz A., Rugeles M.T. (2013). High transcript levels of vitamin D receptor are correlated with higher mRNA expression of human beta defensins and IL-10 in mucosa of HIV-1-exposed seronegative individuals. PLoS ONE.

[19] Yiyan S., Yang S., Li D., Li W. (2022). Vitamin D Affects the Warburg Effect and Stemness Maintenance of Non- Small-Cell Lung Cancer Cells by Regulating the PI3K/AKT/mTOR Signaling Pathway. Curr. Cancer Drug Targets.

[20] Mutt S.J., Raza G.S., Makinen M.J., Keinanen-Kiukaanniemi S., Jarvelin M.R., Herzig K.H. (2020). Vitamin D Deficiency Induces Insulin Resistance and Re-Supplementation Attenuates Hepatic Glucose Output via the PI3K-AKT-FOXO1 Mediated Pathway. Mol. Nutr. Food Res..

[21] Pasquereau S., Herbein G. (2022). CounterAKTing HIV: Toward a “Block and Clear” Strategy?. Front. Cell. Infect. Microbiol..

[22] Beer T.M., Munar M., Henner W.D. (2001). A Phase I trial of pulse calcitriol in patients with refractory malignancies: Pulse dosing permits substantial dose escalation. Cancer.

[23] Fakih M.G., Trump D.L., Muindi J.R., Black J.D., Bernardi R.J., Creaven P.J., Schwartz J., Brattain M.G., Hutson A., French R. (2007). A phase I pharmacokinetic and pharmacodynamic study of intravenous calcitriol in combination with oral gefitinib in patients with advanced solid tumors. Clin. Cancer Res..

[24] Sigmundsdottir H., Pan J., Debes G.F., Alt C., Habtezion A., Soler D., Butcher E.C. (2007). DCs metabolize sunlight-induced vitamin D3 to ’program’ T cell attraction to the epidermal chemokine CCL27. Nat. Immunol..

[25] Fritsche J., Mondal K., Ehrnsperger A., Andreesen R., Kreutz M. (2003). Regulation of 25-hydroxyvitamin D3-1α-hydroxylase and production of 1 α,25-dihydroxyvitamin D3 by human dendritic cells. Blood.

[26] O’Doherty U., Swiggard W.J., Malim M.H. (2000). Human immunodeficiency virus type 1 spinoculation enhances infection through virus binding. J. Virol..

[27] Han H., Cho J.W., Lee S., Yun A., Kim H., Bae D., Yang S., Kim C.Y., Lee M., Kim E. (2018). TRRUST v2: An expanded reference database of human and mouse transcriptional regulatory interactions. Nucleic Acids Res..

[28] Ako-Adjei D., Fu W., Wallin C., Katz K.S., Song G., Darji D., Brister J.R., Ptak R.G., Pruitt K.D. (2015). HIV-1, human interaction database: Current status and new features. Nucleic Acids Res..

[29] Kachalo S., Zhang R., Sontag E., Albert R., DasGupta B. (2008). NET-SYNTHESIS: A software for synthesis, inference and simplification of signal transduction networks. Bioinformatics.

[30] Albert I. Ialbert/Booleannet: Boolean Network Modeling. https://github.com/ialbert/booleannet.

[31] Pozuelo-Rubio M., Leslie N.R., Murphy J., Mackintosh C. (2010). Mechanism of activation of PKB/Akt by the protein phosphatase inhibitor Calyculin A. Cell Biochem. Biophys..

[32] Cheshenko N., Trepanier J.B., Stefanidou M., Buckley N., Gonzalez P., Jacobs W., Herold B.C. (2013). HSV activates Akt to trigger calcium release and promote viral entry: Novel candidate target for treatment and suppression. FASEB J..

[33] Walker N.J. (2002). Tech.Sight. A technique whose time has come. Science.

[34] Szklarczyk D., Kirsch R., Koutrouli M., Nastou K., Mehryary F., Hachilif R., Gable A.L., Fang T., Doncheva N.T., Pyysalo S. (2023). The STRING database in 2023: Protein-protein association networks and functional enrichment analyses for any sequenced genome of interest. Nucleic Acids Res..

[35] Friedrich B.M., Dziuba N., Li G., Endsley M.A., Murray J.L., Ferguson M.R. (2011). Host factors mediating HIV-1 replication. Virus Res..

[36] Baeke F., Korf H., Overbergh L., van Etten E., Verstuyf A., Gysemans C., Mathieu C. (2010). Human T lymphocytes are direct targets of 1,25-dihydroxyvitamin D3 in the immune system. J. Steroid Biochem. Mol. Biol..

[37] Gonzalez S.M., Aguilar-Jimenez W., Trujillo-Gil E., Zapata W., Su R.C., Ball T.B., Rugeles M.T. (2019). Vitamin D treatment of peripheral blood mononuclear cells modulated immune activation and reduced susceptibility to HIV-1 infection of CD4+ T lymphocytes. PLoS ONE.

[38] Abdullah L., Hills L.B., Winter E.B., Huang Y.H. (2021). Diverse Roles of Akt in T cells. Immunometabolism.

[39] Maira S.M., Galetic I., Brazil D.P., Kaech S., Ingley E., Thelen M., Hemmings B.A. (2001). Carboxyl-terminal modulator protein (CTMP), a negative regulator of PKB/Akt and v-Akt at the plasma membrane. Science.

[40] Wang Q., He Y., Shen Y., Zhang Q., Chen D., Zuo C., Qin J., Wang H., Wang J., Yu Y. (2014). Vitamin D inhibits COX-2 expression and inflammatory response by targeting thioesterase superfamily member 4. J. Biol. Chem..

[41] Pearce E.L., Poffenberger M.C., Chang C.H., Jones R.G. (2013). Fueling immunity: Insights into metabolism and lymphocyte function. Science.

[42] Pan X., Baldauf H.M., Keppler O.T., Fackler O.T. (2013). Restrictions to HIV-1 replication in resting CD4+ T lymphocytes. Cell Res..

[43] Lever A.M., Jeang K.T. (2011). Insights into cellular factors that regulate HIV-1 replication in human cells. Biochemistry.

[44] Liu S., Liao S., Liang L., Deng J., Zhou Y. (2023). The relationship between CD4(+) T cell glycolysis and their functions. Trends Endocrinol. Metab..

[45] Palmer C.S., Ostrowski M., Gouillou M., Tsai L., Yu D., Zhou J., Henstridge D.C., Maisa A., Hearps A.C., Lewin S.R. (2014). Increased glucose metabolic activity is associated with CD4+ T-cell activation and depletion during chronic HIV infection. AIDS.

[46] Kato H., Takita J., Miyazaki T., Nakajima M., Fukai Y., Masuda N., Fukuchi M., Manda R., Ojima H., Tsukada K. (2002). Glut-1 glucose transporter expression in esophageal squamous cell carcinoma is associated with tumor aggressiveness. Anticancer Res..

[47] Xiang J., Wang K., Tang N. (2023). PCK1 dysregulation in cancer: Metabolic reprogramming, oncogenic activation, and therapeutic opportunities. Genes Dis..

[48] Ho P.C., Bihuniak J.D., Macintyre A.N., Staron M., Liu X., Amezquita R., Tsui Y.C., Cui G., Micevic G., Perales J.C. (2015). Phosphoenolpyruvate Is a Metabolic Checkpoint of Anti-tumor T Cell Responses. Cell.

[49] Toledano Zur R., Atar O., Barliya T., Hoogi S., Abramovich I., Gottlieb E., Ron-Harel N., Cohen C.J. (2024). Genetically engineering glycolysis in T cells increases their antitumor function. J. Immunother. Cancer.

[50] Guo M., Abd-Rabbo D., Bertol B.C., Carew M., Lukhele S., Snell L.M., Xu W., Boukhaled G.M., Elsaesser H., Halaby M.J. (2023). Molecular, metabolic, and functional CD4 T cell paralysis in the lymph node impedes tumor control. Cell Rep..

[51] Fisher S.A., Rahimzadeh M., Brierley C., Gration B., Doree C., Kimber C.E., Plaza Cajide A., Lamikanra A.A., Roberts D.J. (2019). The role of vitamin D in increasing circulating T regulatory cell numbers and modulating T regulatory cell phenotypes in patients with inflammatory disease or in healthy volunteers: A systematic review. PLoS ONE.

[52] Pompura S.L., Dominguez-Villar M. (2018). The PI3K/AKT signaling pathway in regulatory T-cell development, stability, and function. J. Leukoc. Biol..

[53] Hawse W.F., Boggess W.C., Morel P.A. (2017). TCR Signal Strength Regulates Akt Substrate Specificity To Induce Alternate Murine Th and T Regulatory Cell Differentiation Programs. J. Immunol..

[54] Gerriets V.A., Kishton R.J., Johnson M.O., Cohen S., Siska P.J., Nichols A.G., Warmoes M.O., de Cubas A.A., MacIver N.J., Locasale J.W. (2016). Foxp3 and Toll-like receptor signaling balance T(reg) cell anabolic metabolism for suppression. Nat. Immunol..

[55] Macintyre A.N., Gerriets V.A., Nichols A.G., Michalek R.D., Rudolph M.C., Deoliveira D., Anderson S.M., Abel E.D., Chen B.J., Hale L.P. (2014). The glucose transporter Glut1 is selectively essential for CD4 T cell activation and effector function. Cell Metab..

[56] Kempkes R.W.M., Joosten I., Koenen H., He X. (2019). Metabolic Pathways Involved in Regulatory T Cell Functionality. Front. Immunol..

[57] Steele L., Mannion A.J., Shaw G., Maclennan K.A., Cook G.P., Rudd C.E., Taylor A. (2021). Non-redundant activity of GSK-3α and GSK-3β in T cell-mediated tumor rejection. iScience.

[58] Jendrossek V., Henkel M., Hennenlotter J., Vogel U., Ganswindt U., Muller I., Handrick R., Anastasiadis A.G., Kuczyk M., Stenzl A. (2008). Analysis of complex protein kinase B signalling pathways in human prostate cancer samples. BJU Int..

[59] Xia Y., Zhuo H., Lu Y., Deng L., Jiang R., Zhang L., Zhu Q., Pu L., Wang X., Lu L. (2015). Glycogen synthase kinase 3β inhibition promotes human iTreg differentiation and suppressive function. Immunol. Res..

[60] Wang R., Dillon C.P., Shi L.Z., Milasta S., Carter R., Finkelstein D., McCormick L.L., Fitzgerald P., Chi H., Munger J. (2011). The transcription factor Myc controls metabolic reprogramming upon T lymphocyte activation. Immunity.

[61] Garcia-Gutierrez L., Bretones G., Molina E., Arechaga I., Symonds C., Acosta J.C., Blanco R., Fernandez A., Alonso L., Sicinski P. (2019). Myc stimulates cell cycle progression through the activation of Cdk1 and phosphorylation of p27. Sci. Rep..

[62] Taniguchi C.M., Emanuelli B., Kahn C.R. (2006). Critical nodes in signalling pathways: Insights into insulin action. Nat. Rev. Mol. Cell Biol..

[63] Lan X., Cheng K., Chandel N., Lederman R., Jhaveri A., Husain M., Malhotra A., Singhal P.C. (2013). High glucose enhances HIV entry into T cells through upregulation of CXCR4. J. Leukoc. Biol..

[64] Fujinaga K., Huang F., Peterlin B.M. (2023). P-TEFb: The master regulator of transcription elongation. Mol. Cell.

[65] Hafer T.L., Felton A., Delgado Y., Srinivasan H., Emerman M. (2023). A CRISPR Screen of HIV Dependency Factors Reveals That CCNT1 Is Non-Essential in T Cells but Required for HIV-1 Reactivation from Latency. Viruses.

[66] Mbonye U., Karn J. (2024). The cell biology of HIV-1 latency and rebound. Retrovirology.

[67] Rice A.P., Herrmann C.H. (2003). Regulation of TAK/P-TEFb in CD4+ T lymphocytes and macrophages. Curr. HIV Res..

[68] Ellegard R., Shankar E.M., Larsson M. (2011). Targeting HIV-1 innate immune responses therapeutically. Curr. Opin. HIV AIDS.

[69] Ruffin N., Brezar V., Ayinde D., Lefebvre C., Schulze Zur Wiesch J., van Lunzen J., Bockhorn M., Schwartz O., Hocini H., Lelievre J.D. (2015). Low SAMHD1 expression following T-cell activation and proliferation renders CD4+ T cells susceptible to HIV-1. AIDS.

[70] Blanco R., Gomez de Cedron M., Gamez-Reche L., Martin-Leal A., Gonzalez-Martin A., Lacalle R.A., Ramirez de Molina A., Manes S. (2021). The Chemokine Receptor CCR5 Links Memory CD4(+) T Cell Metabolism to T Cell Antigen Receptor Nanoclustering. Front. Immunol..

[71] Jacobs E.S., Keating S.M., Abdel-Mohsen M., Gibb S.L., Heitman J.W., Inglis H.C., Martin J.N., Zhang J., Kaidarova Z., Deng X. (2017). Cytokines Elevated in HIV Elite Controllers Reduce HIV Replication In Vitro and Modulate HIV Restriction Factor Expression. J. Virol..

[72] Schwab J.D., Kuhlwein S.D., Ikonomi N., Kuhl M., Kestler H.A. (2020). Concepts in Boolean network modeling: What do they all mean?. Comput. Struct. Biotechnol. J..

[73] Oertelt-Prigione S. (2012). Immunology and the menstrual cycle. Autoimmun. Rev..

[74] Alanazi H., Zhang Y., Fatunbi J., Luu T., Kwak-Kim J. (2024). The impact of reproductive hormones on T cell immunity; normal and assisted reproductive cycles. J. Reprod. Immunol..

