# Supplementary material for: Vitamin D Decreases Susceptibility of CD4+ T Cells to HIV Infection by Reducing AKT Phosphorylation and Glucose Uptake: A Bioinformatic and In Vitro Approach
Source: Biomolecules. 2025 Mar 18;15(3):432. doi: 10.3390/biom15030432 (PMC11940553; doi:10.3390/biom15030432)

**Supplemental Figure S1.** Purity and Viability of negatively selected CD4^+^ T Cells used for *in vitro* assays.


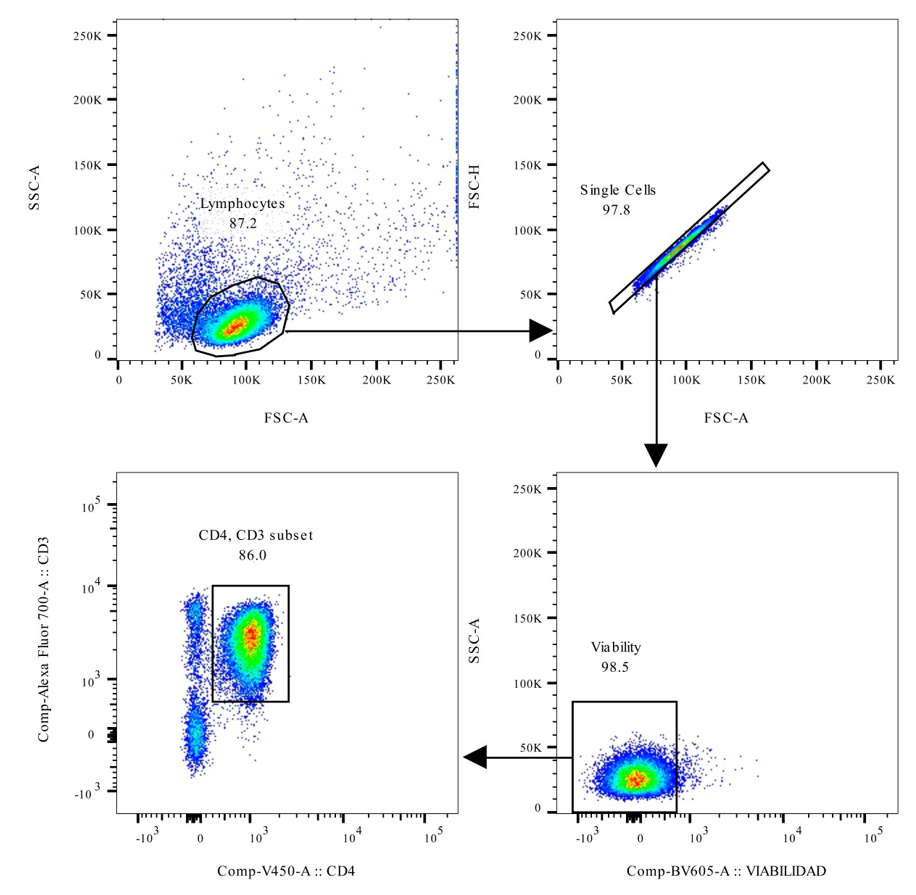


**Supplementary Figure S2. Simplified gene regulatory network of interactions relevant to HIV infection.** The initial network, consisting of 4932 interactions, was refined to 1302 through Net-Synthesis, which removed redundancies while preserving critical regulatory relationships. This simplification enhanced the interpretability of the network and optimized it for computational analysis. The network depicts undirected interactions with nodes representing genes and edges denoting regulatory relationships, classified as activation or inhibition.

**Supplementary Figure S3. Heatmap of nodes states across 40 simulation steps in the gene regulatory network.** The dynamic behavior of each node is displayed at time t+1 determined by Boolean expressions based on the states of its regulatory inputs at time t. The asynchronous update setting ensures that each node is updated once per time unit in a predefined order, simulating the sequential progression of regulatory events. The leftmost column for each scenario represents the initial states of all nodes (arrows), while the rightmost column corresponds to their states at the final simulation step (t=40).

**Figure S4.** **Gating strategy to determine the effect of VitD on AKT phosphorylation in CD4^+^ T cells stimulated with 100 nM Calyculin A and treated with VitD, EtOH, or with Miltefosine (15 µM), a known AKT inhibitor, as a negative control.** Compensation of fluorochrome spillover was achieved using unstained and single-stained cells for each antibody. An unstimulated control (without Calyculin or any treatment) was also included to establish a pAKT phosphorylation baseline. The pseudocolor dotplots of the gating used in the analysis strategy are shown, along with a histogram overlaying the evaluated conditions.


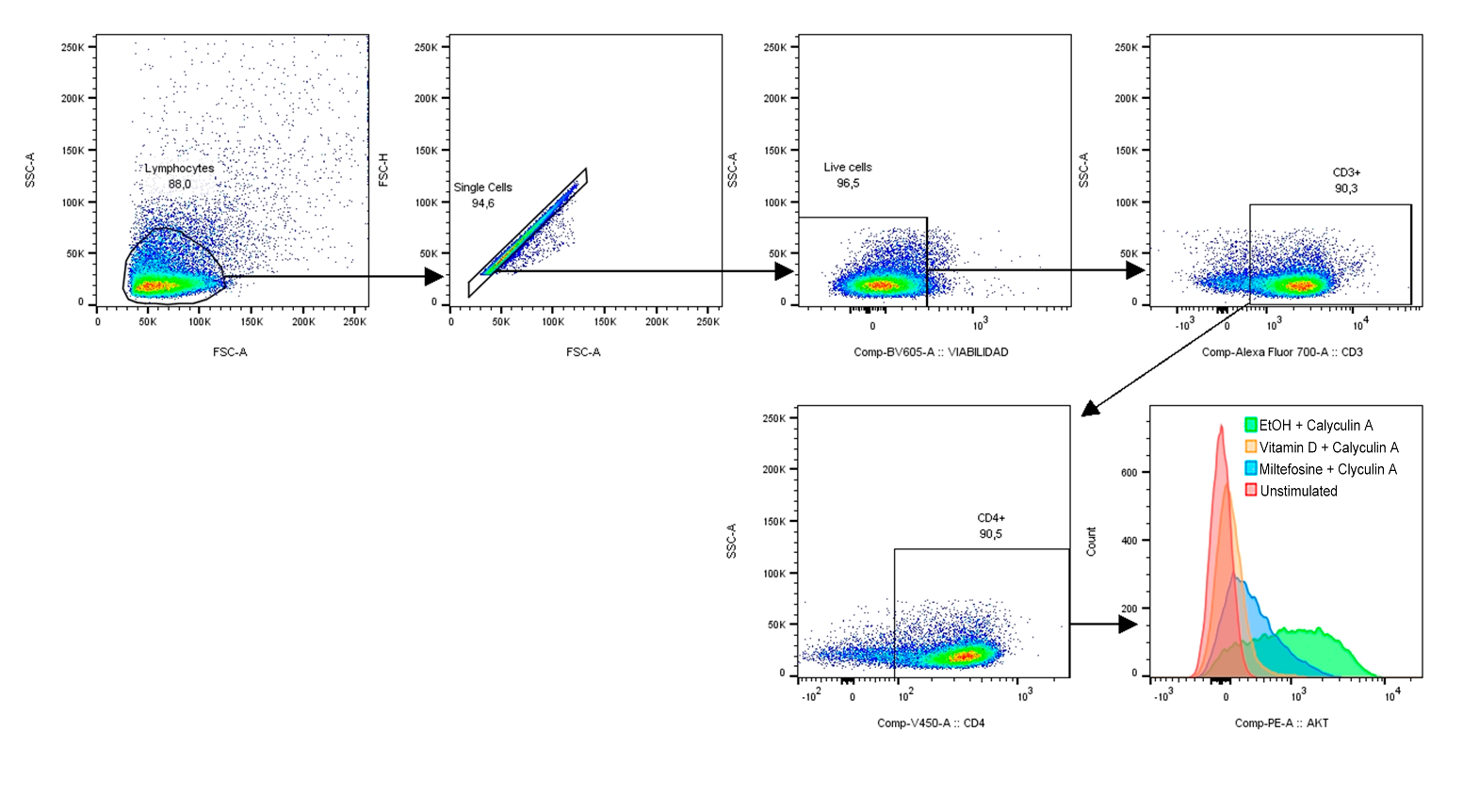


**Supplemental Figure S5.** **Effect of VitD on the expression of other evaluated proviral and antiviral genes in CD4+ T cells treated with VitD or EtOH for 16 hours.** **A.** Expression of the VDR gene, a key effector in vitamin D metabolism and VitD-associated gene regulation. **B.** Expression of SAMHD1 gene, an antiviral gene involved in the immune response. **C.** Expression of ADAM10 gene, a proviral gene involved in the proteolysis of transmembrane proteins and regulation of cellular signaling. **D.** Expression of PLD1 gene, a proviral gene involved in the hydrolysis of phosphatidylcholine and signal transduction.


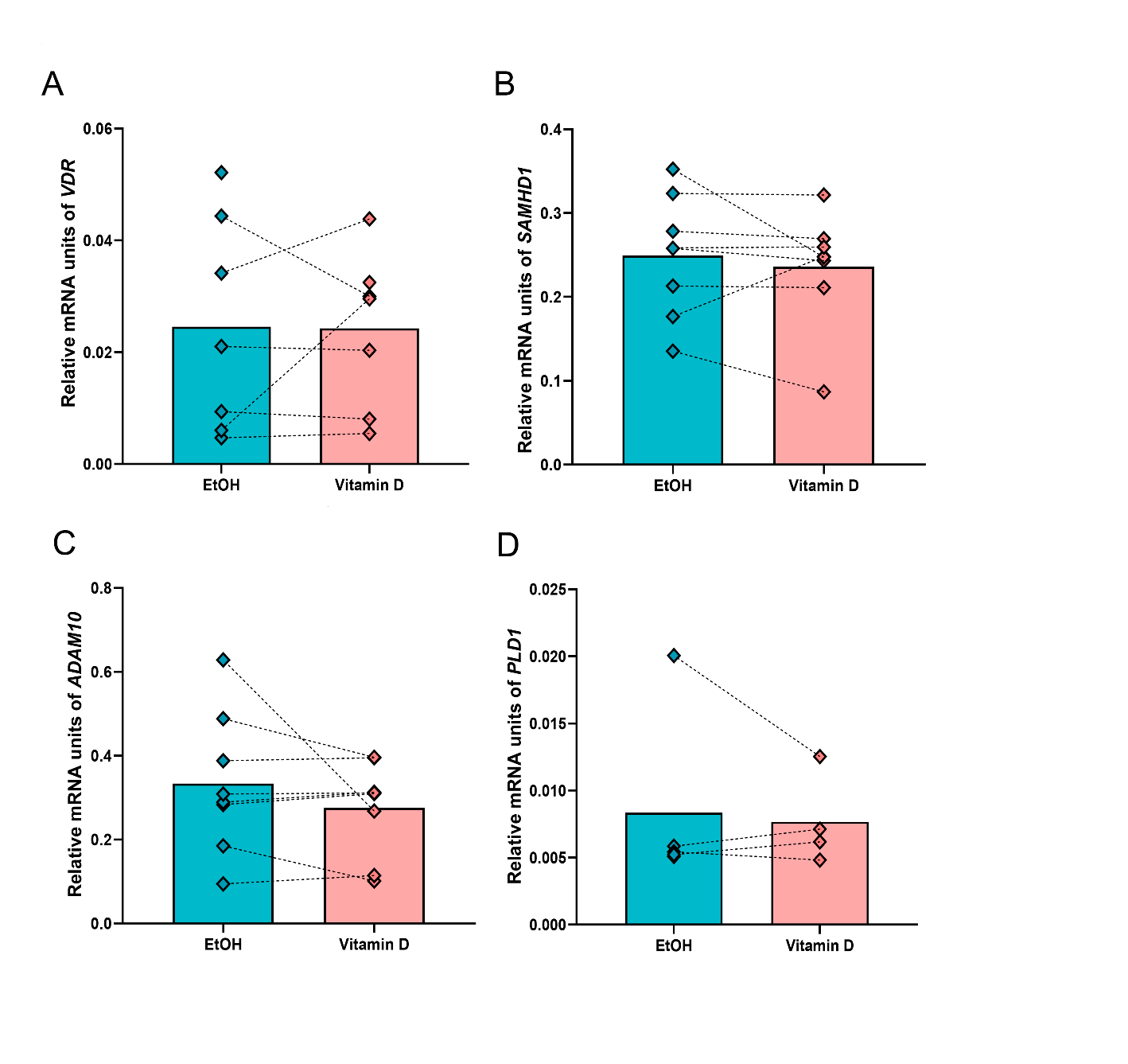


**Supplemental Figure S6.** **Effect of Vit D on the expression of CXCR4 and CCR5 coreceptors in CD4+ T cells treated with VitD or EtOH for 16 hours. MFI of CXCR4 and CCR5 under VitD, calyculin and miltefosine stimulation.** Statistical comparisons were performed using Wilcoxon matched pairs signed rank test. "The expression of coreceptors CXCR4 and CCR5 was measured using flow cytometry, with CXCR4 detected in the APC-Cy7 channel and CCR5 in the PE-Cy5 channel, respectively.

**
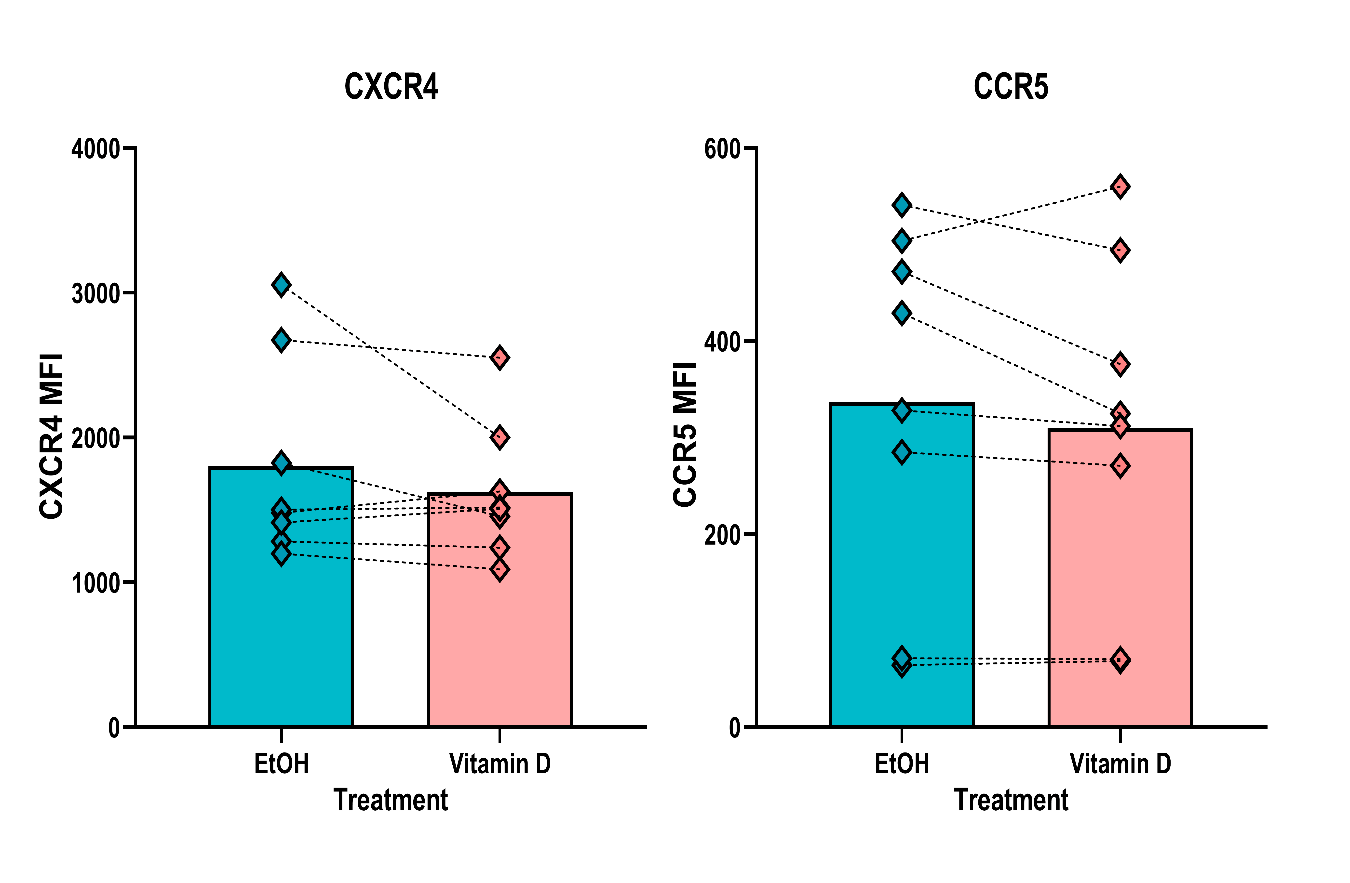
**

**Supplemental Figure S7.** **Viability of CD4 T cell under Vit D and EtOH treatments.** A. Viability of CD4 T cells in the presence of Vit D or EtOH for 16 and 72 hours. Viability was assessed in PBMCs with or without PHA, using Live/Dead Fixable Yellow Dead Cell Stain (Invitrogen™) by flow cytometry, following the exclusion of aggregates (FSC-H vs FSC-A). **B.** Viability of CD4 T cells in the presence of Vit D or EtOH for 16 hours in CD4+ T cells stimulated with 100 nM Calyculin A and treated with Vit D or EtOH, using Live/Dead Fixable Yellow Dead Cell Stain (Invitrogen™) by flow cytometry (n=5).


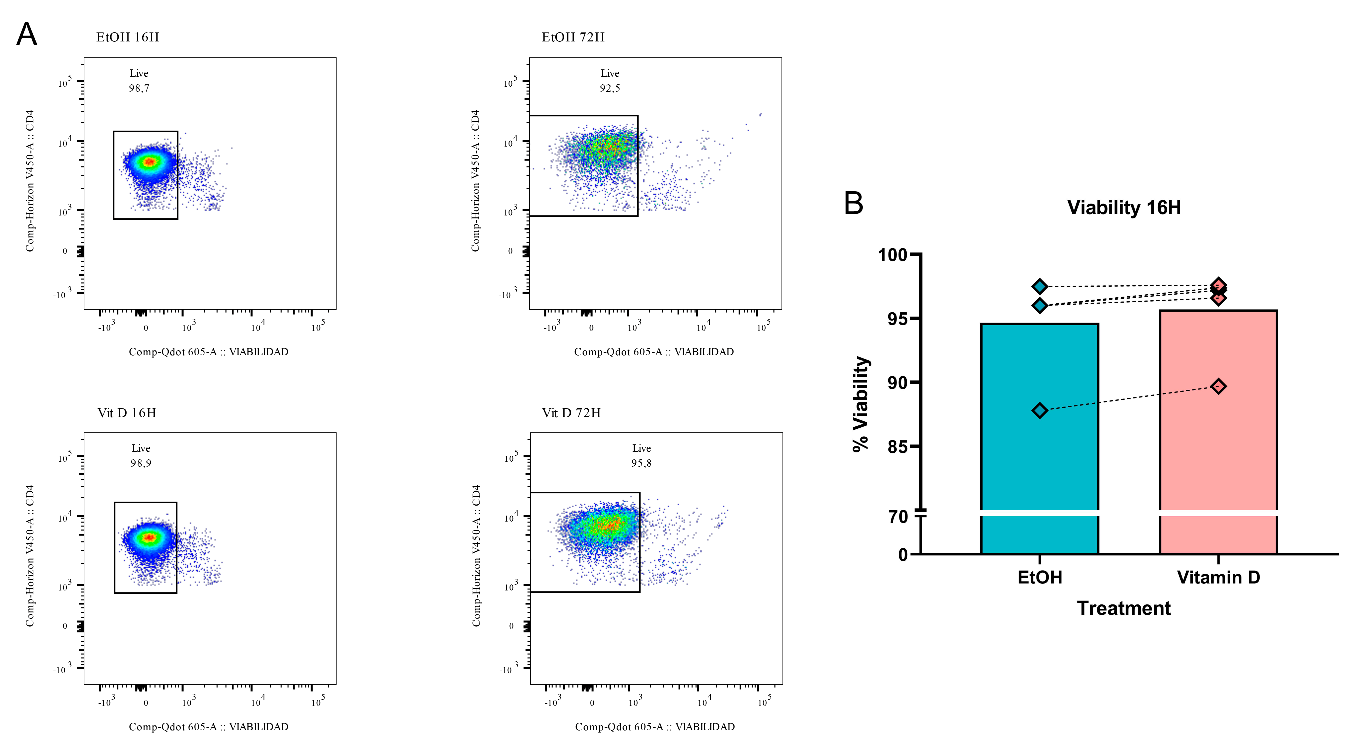

Supplement: Supplementary file 1 [file biomolecules-15-00432-s001.zip › Figuras y datos suplementarios art/Supplementary Figures (with Legend).docx]
