# Supplementary figures and images for: Vitamin D Decreases Susceptibility of CD4+ T Cells to HIV Infection by Reducing AKT Phosphorylation and Glucose Uptake: A Bioinformatic and In Vitro Approach
Source: Biomolecules. 2025 Mar 18;15(3):432. doi: 10.3390/biom15030432 (PMC11940553; doi:10.3390/biom15030432)

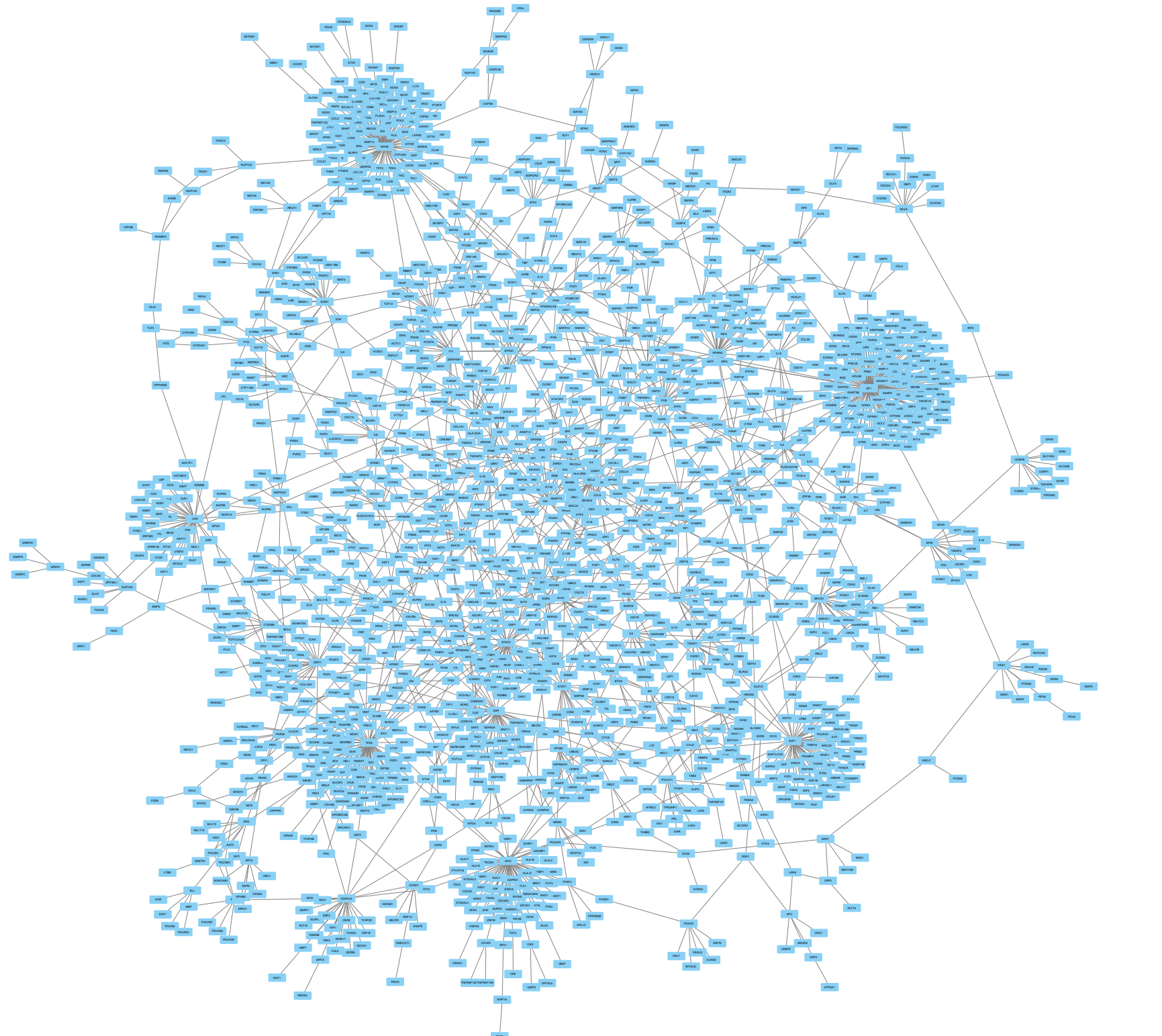

Supplement: Supplementary file 1 [file biomolecules-15-00432-s001.zip › Figuras y datos suplementarios art/Fig S2. Simplified network.pdf]

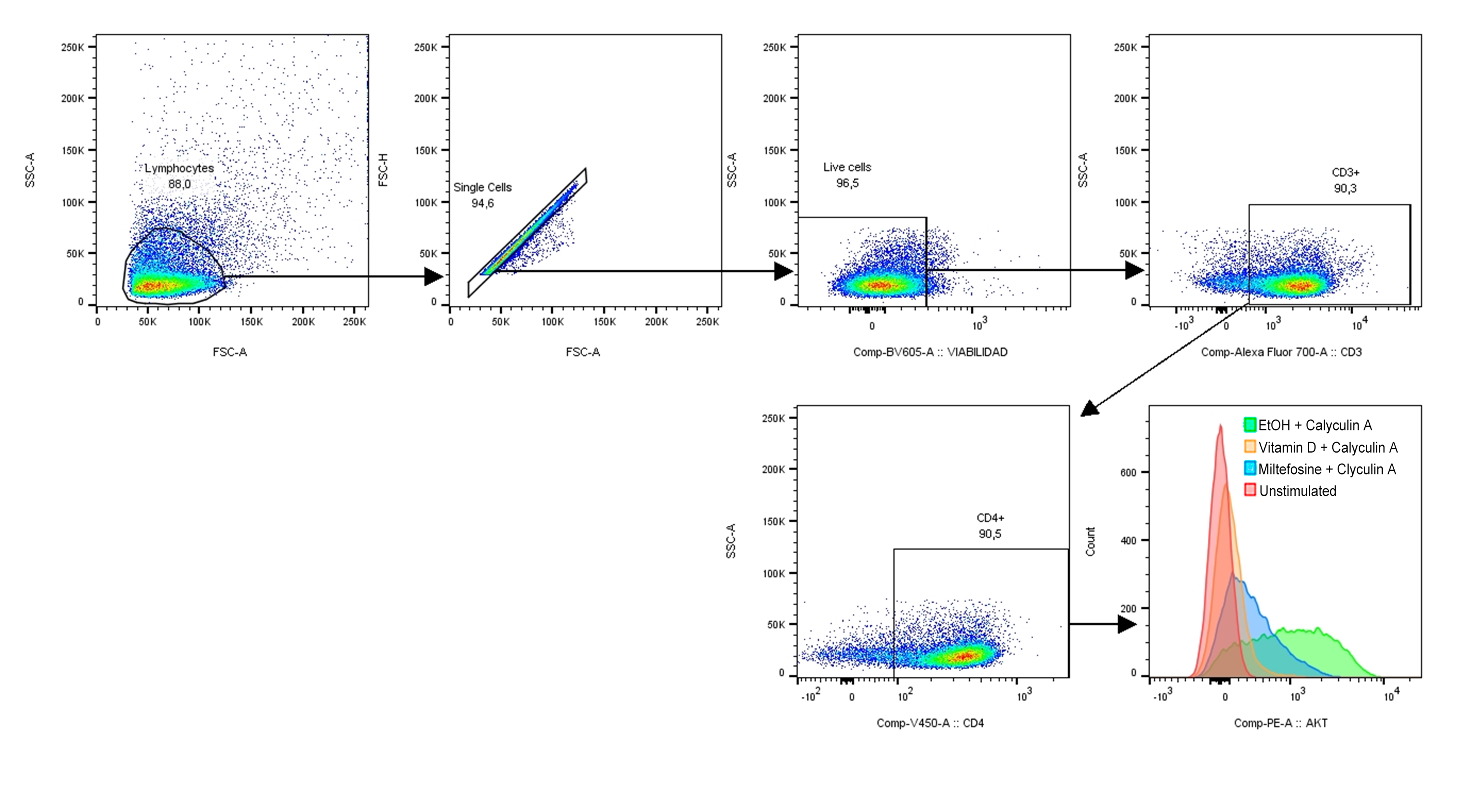

Supplement: Supplementary file 1 [file biomolecules-15-00432-s001.zip › Figuras y datos suplementarios art/Fig S4. Gating strategy to determine the effect of VitD on AKT phosphorylation.tif]

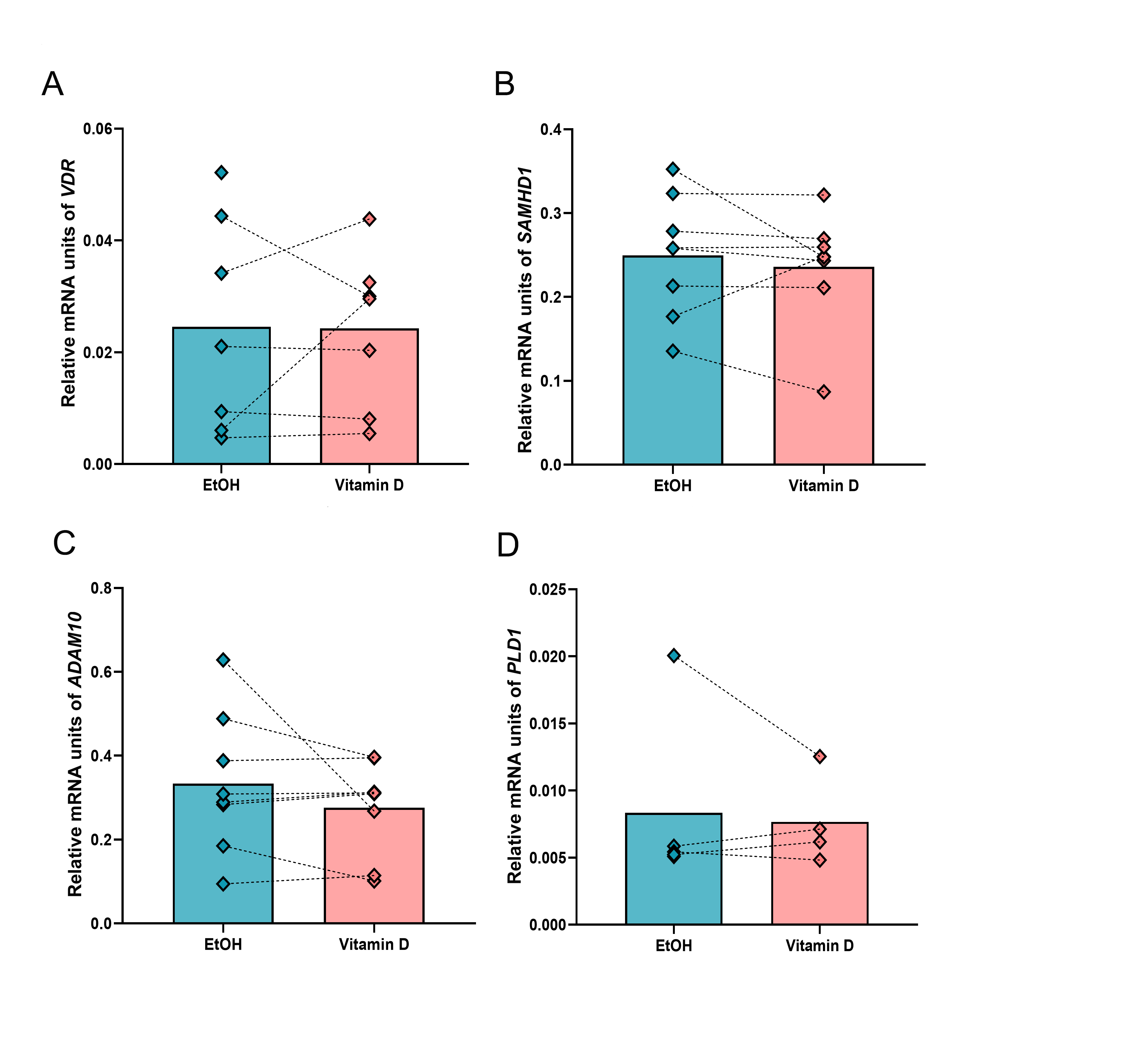

Supplement: Supplementary file 1 [file biomolecules-15-00432-s001.zip › Figuras y datos suplementarios art/Fig S5. qPCR results of genes not modulated by VitD.tif]

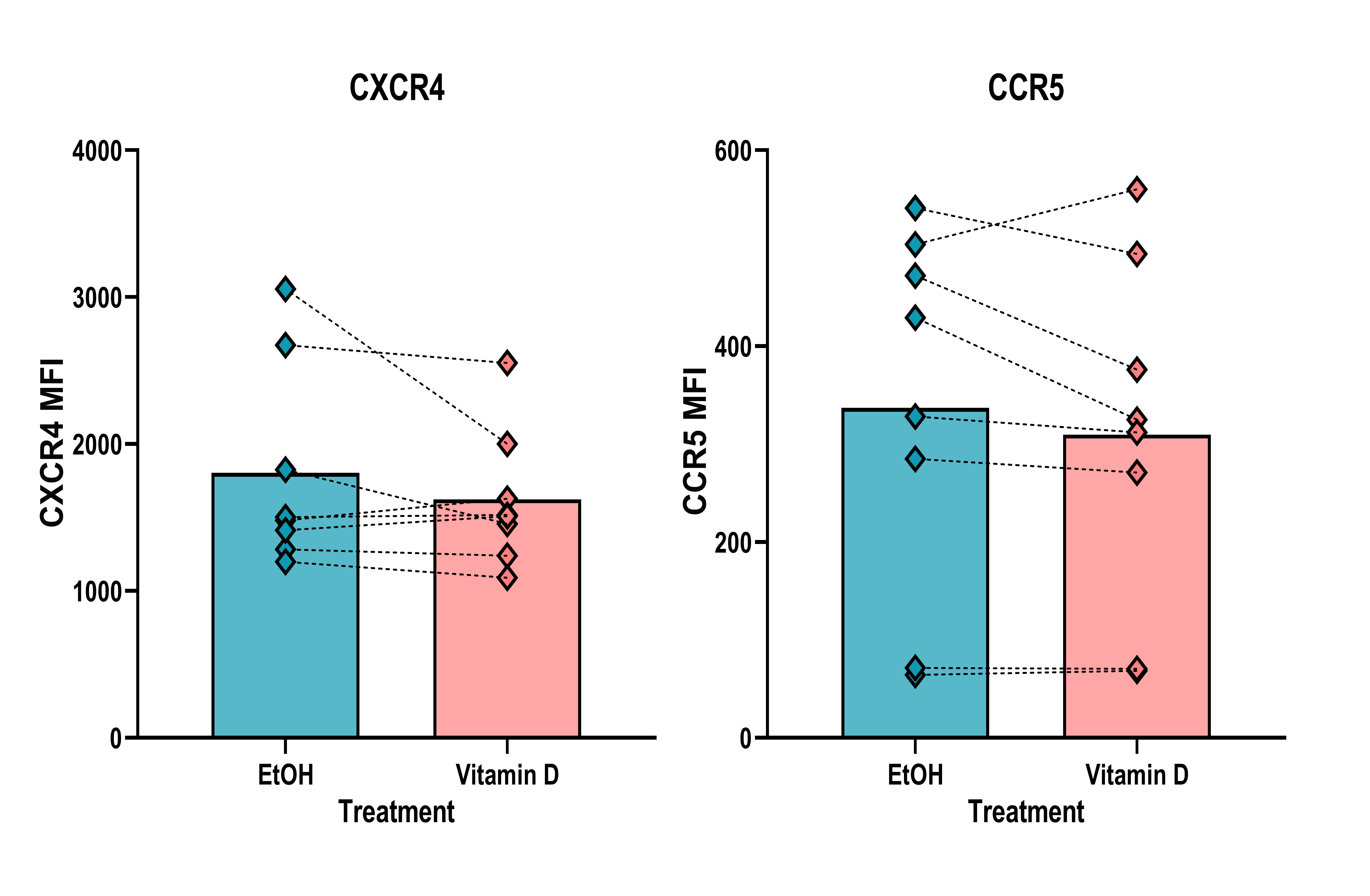

Supplement: Supplementary file 1 [file biomolecules-15-00432-s001.zip › Figuras y datos suplementarios art/Fig S6. Effect of VitD on the expression of CXCR4 and CCR5 coreceptors.tif]

# Boolean network simulation states

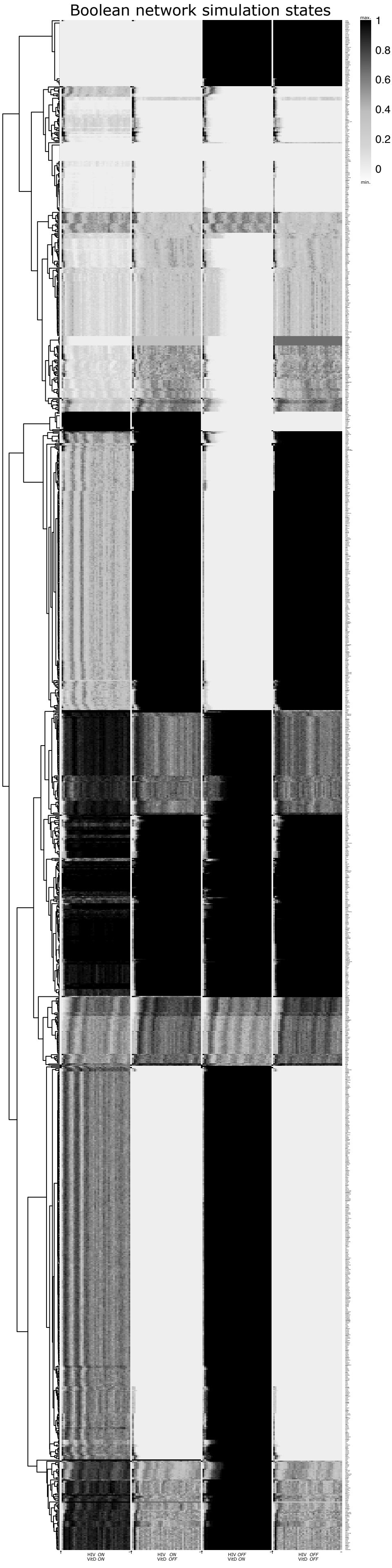

Supplement: Supplementary file 1 [file biomolecules-15-00432-s001.zip › Figuras y datos suplementarios art/Fig S3. Heatmap visualization of all genes following 40 simulation steps.pdf]

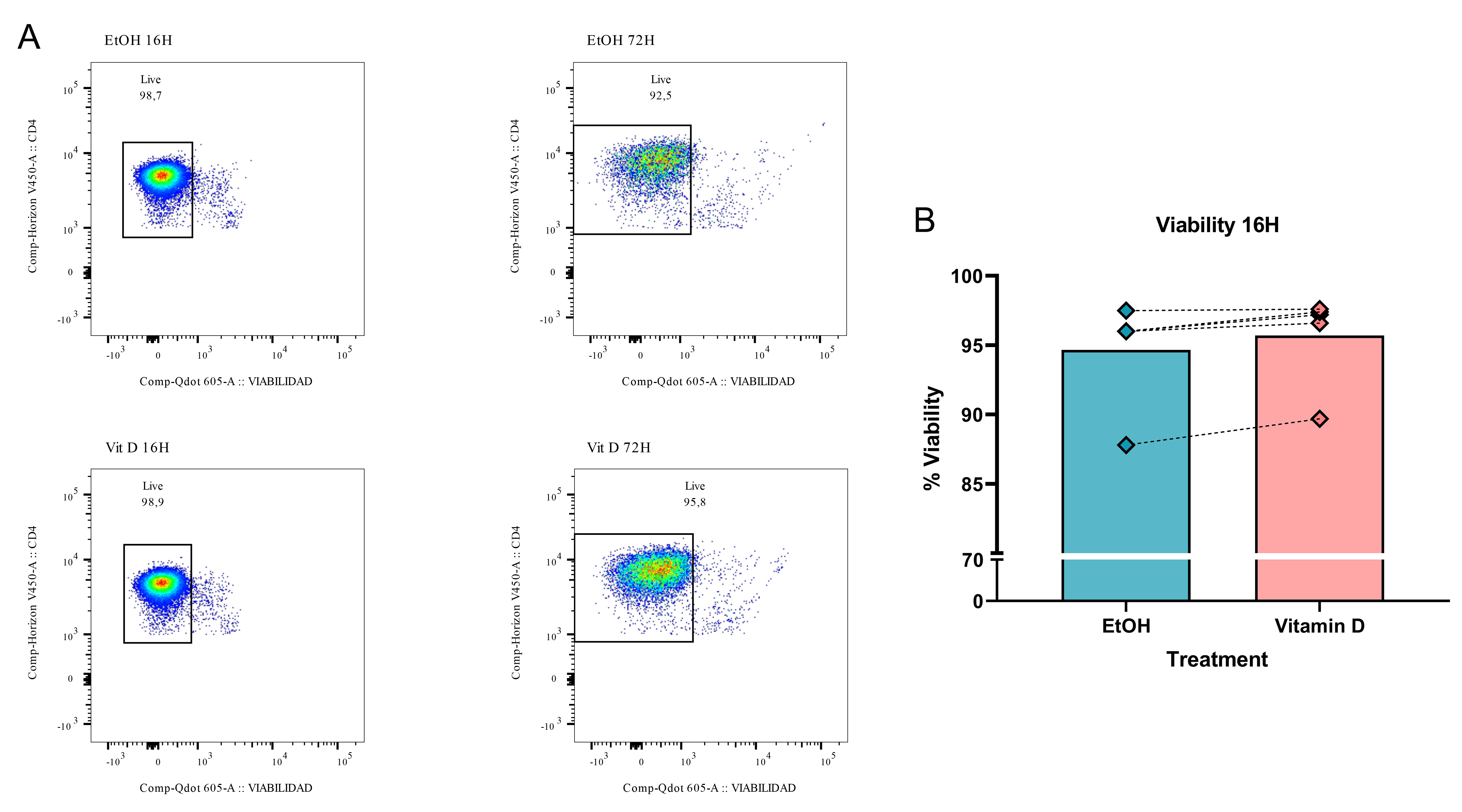

Supplement: Supplementary file 1 [file biomolecules-15-00432-s001.zip › Figuras y datos suplementarios art/Fig S7. Viability of CD4 T cell under Vitamin D and EtOH treatments.tif]
